# Indication of Thalamo-Cortical Circuit Dysfunction in Idiopathic Normal Pressure Hydrocephalus: A Tensor Imaging Study

**DOI:** 10.1038/s41598-020-63238-7

**Published:** 2020-04-09

**Authors:** Andreas Eleftheriou, Ida Blystad, Anders Tisell, Johan Gasslander, Fredrik Lundin

**Affiliations:** 1 Department of Neurology in Linköping, and Department of Biomedical and Clinical Sciences, Linköping University, Linköping, Sweden; 2 Department of Radiology in Linköping, and Department of Health, Medicine and Caring Sciences, Linköping University, Linköping, Sweden; 3 Department of Medical Radiation Physics, and Department of Health, Medicine and Caring Sciences, Linköping University, Linköping, Sweden; 4 Center for Medical Image Science and Visualisation (CMIV), Linköping University, Linköping, Sweden; 5 Department of Cardiology and Department of Health, Medicine and Caring Sciences, Linköping University, Norrkoping, Sweden

**Keywords:** Hydrocephalus, Fluid dynamics

## Abstract

Idiopathic normal pressure hydrocephalus (iNPH) is a disorder with unclear pathophysiology. The diagnosis of iNPH is challenging due to its radiological similarity with other neurodegenerative diseases and ischemic subcortical white matter changes. By using Diffusion Tensor Imaging (DTI) we explored differences in apparent diffusion coefficient (ADC) and fractional anisotropy (FA) in iNPH patients (before and after a shunt surgery) and healthy individuals (HI) and we correlated the clinical results with DTI parameters. Thirteen consecutive iNPH-patients underwent a pre- and post-operative clinical work-up: 10 m walk time (w10mt) steps (w10ms), TUG-time (TUGt) and steps (TUGs); for cognitive function MMSE. Nine HI were included. DTI was performed before and 3 months after surgery, HI underwent DTI once. DTI differences analyzed by manually placing 12 regions-of-interest. In patients motor and balance function improved significantly after surgery (p = 0.01, p = 0.025). Higher nearly significant FA values found in the patients vs HI pre-operatively in the thalamus (p = 0.07) accompanied by an almost significant lower ADC (p = 0.08). Significantly FA and ADC-values were found between patients and HI in FWM (p = 0.02, p = 0.001) and almost significant (p = 0.057) pre- vs postoperatively. Postoperatively we found a trend towards the HIs FA values and a strong significant negative correlation between FA changes vs. gait results in the FWM (r = −0.7, p = 0.008). Our study gives a clear indication of an ongoing pathological process in the periventricular white matter, especially in the thalamus and in the frontal white matter supporting the hypothesis of a shunt reversible thalamo-cortical circuit dysfunction in iNPH.

## Introduction

Idiopathic normal pressure hydrocephalus (iNPH) is a disorder that features disturbance of gait and balance, cognitive decline and urinary incontinence caused by impaired turnover of cerebrospinal fluid (CSF)^1^. In this disorder, however, the CSF opening pressure upon lumbar puncture is within the normal range^2^. Brain imaging in typical cases shows ventriculomegaly with an Evans’ index >0.3, enlarged Sylvian fissures, tight medial and high convexity sulci, callosal angle between <50° and >90° and disproportionately enlarged subarachnoid-space hydrocephalus (DESH)^3–5^. iNPH can be identified by a combination of radiological findings and clinical features, but due to similarities between iNPH and other neurodegenerative diseases, such as Alzheimer´s disease (AD), subcortical vascular encephalopathy, and various types of atypical parkinsonism, diagnosis can be difficult^6^. Thus, not all patients who meet the criteria for iNPH improve after surgical intervention^7^.

Cerebral blood flow (CBF) studies have revealed reduced perfusion in the immediate periventricular white matter compared to the subcortical white matter in iNPH patients^8,9^. The periventricular brain tissue is also characterised by neuronal degeneration and gliosis, presumably caused by modified extracellular fluid dynamics^10^. Periventricular hyperintensities visualised with magnetic resonance imaging (MRI) may exist both in iNPH and in subcortical vascular encephalopathy (Binswanger’s disease). These diseases may be present simultaneously and can contribute to the symptomatology in different degrees. There is an urgent need for a better prediction of positive shunt responsiveness. Consequently, there is increasing interest in studying white matter changes in iNPH by using diffusion tensor imaging (DTI)^11^. The combination of 2D diffusion-weighted images, including diagonal elements, in a 3D diffusion assessment creates a high-resolution MR technique that can reveal the integrity of periventricular white matter changes^12^. DTI integrity changes are quantified by the apparent diffusion coefficient (ADC), which shows the diffusion changes, and by the fractional anisotropy (FA), indicating the directivity of the ADC^13^. Increased FA may be caused by, for example, compression of white matter^14^, and decreased FA is associated with axonal degeneration, brain oedema or both^15^.

The primary aim of this study was to identify differences in DTI parameters (ADC and FA) in patients with iNPH, both before and after shunt surgery, as well as in healthy individuals (HIs). The secondary aim was to determine whether there was any correlation between clinical symptoms and DTI parameters.

## Participants and Methods

### Patients

The study included thirteen consecutive patients referred to the Department of Neurology, University Hospital of Linköping. The subjects were six males and seven females with a median age of 75 (range 49–81) years who fulfilled the diagnostic criteria for probable iNPH according to the international guidelines of iNPH^16^. The clinical characteristics of the patients are displayed in Table 1.

**Table 1:** Clinical characteristics of the iNPH patients and HIs.

Demography and Comorbidity		
Category	iNPH (n = 13)	HIs (n = 9)
*Male/female*	6/7	4/5
*Age, median (range), years*	75 (49–81)	77 (70–90)
BMI (male/female), median	27/28	23/21
Diabetes mellitus	1	1
Atrial fibrillation	1	1
Hypertension	6	1
Intermittent claudication	1	0
Stroke	1	0
Polyneuropathy	2	0
Chronic ischaemic heart disease	4	0

The study was approved by the Regional Ethical Review Board of Linköping, Sweden (Dnr M11–07). All participants gave written consent. The study was performed according to the Declaration of Helsinki. Informed written consent was obtained from the patients and HIs, according to the Declaration of Helsinki.

In accordance with the routine of our department, all patients underwent a clinical examination by a neurologist. A motor evaluation (10 m walk time (w10mt), 10 m walk steps (w10ms), timed up and go time (TUG-t), timed up and go steps (TUG-s), and Romberg’s test) was performed by a physiotherapist, and cognitive testing (Mini Mental State Examination (MMSE) and Trail Making A test (TMT-A) were performed by an occupational therapist. All patients underwent a CSF tap test to select suitable candidates for a shunt operation. Eligible patients received a ventriculo-peritoneal shunt, and the motor and cognitive evaluations were repeated three months after the operation.

### HIs

Nine convenience-sampled HIs, comprising four males and five females with a median age of 77 (range 70–90) years, were included. None of them was diagnosed with a neurological disease before or during the study period. All HIs underwent a motor evaluation (w10ms, w10mt and Romberg’s test) and a cognitive examination (MMSE) at a single timepoint.

### DTI

DTI was performed before and three months after shunt surgery, and HIs underwent DTI once.

All MRI examinations were carried out at the Center for Medical Image Science and Visualisation (CMIV) using a 1.5 Tesla Achieva MR system (Philips Healthcare, Best) with an eight-channel phased-array head coil. DTI volumes were acquired with two averages at b value 0 and 15 direction with b value 1000, field-of-view (FOV) 240 *240 * 90, acquired resolution 3*3*3 cubic millimetres (mm^3^), reconstructed resolution 1.9*1.9*3 mm^3^, echo time (TE) 90 milliseconds (ms), and repetition time (TR) 4 s. ADC and FA were calculated using the IntelliSpace portal Philips Medical, Best. Regions of interest (ROIs) were manually drawn on DTI images with b values of 0 using a program developed in house and produced by MevisLab (MeVis Medical Solution AG, Germany).

**Figure 1:**
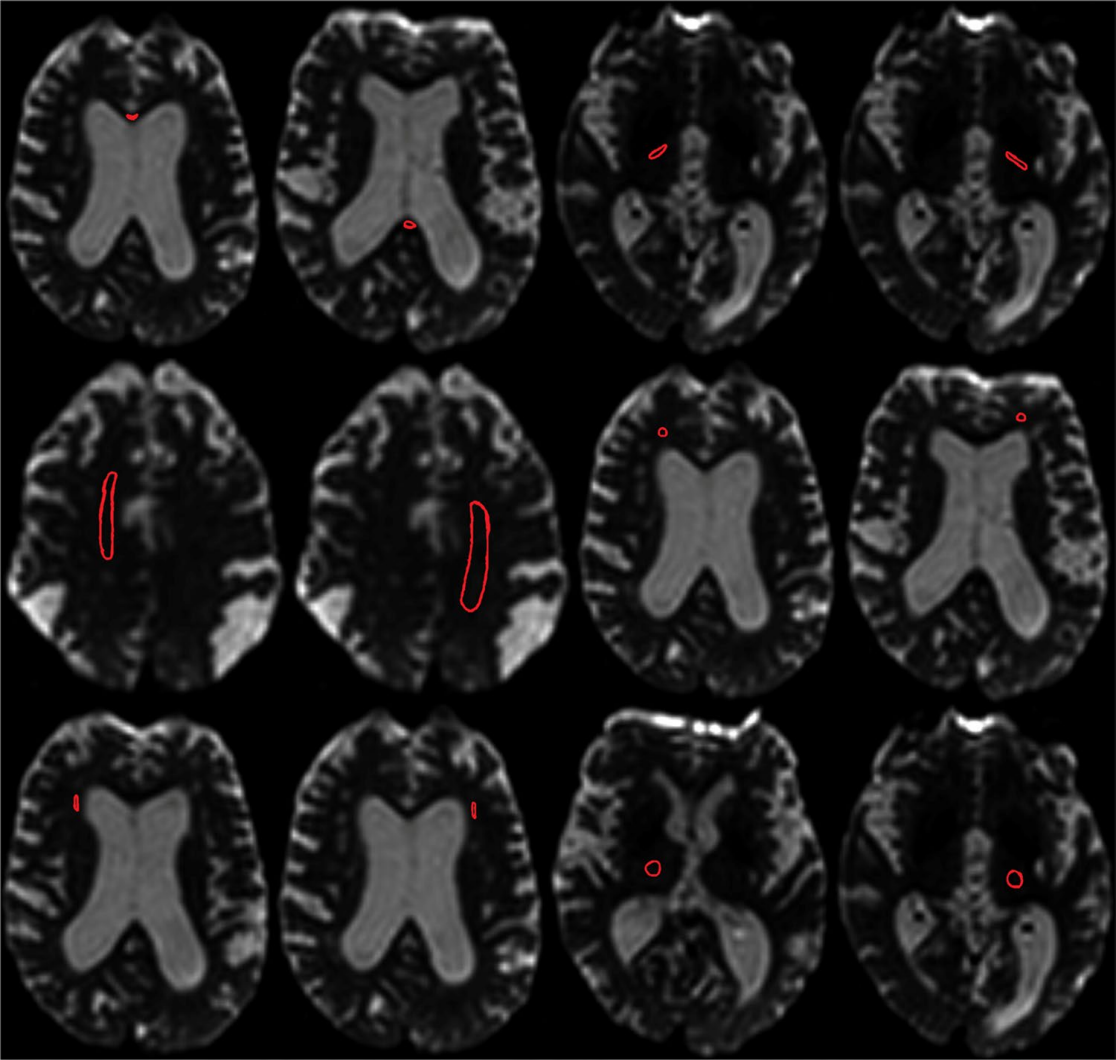
12 ROIs along the corpus callosum (CC) (genu and splenium), capsula interna right and left (CIdx, CIsin), centrum semiovale right and left (CSdx, CSsin), one in the frontal white matter right and left (FWMdx, FWMsin) one in the lateral frontal white matter right and left (LWMdx, LWMsin) and one in the thalamus right and left (THdx, THsin).

ADC differences were analysed by manually placing 12 ROIs along the corpus callosum (CC) (genu and splenium), right and left capsula interna (CIdx, CIsin), right and left centrum semiovale (CSdx, CSsin), right and left frontal white matter (FWMdx, FWMsin), right and left lateral frontal white matter (LWMdx, LWMsin), and right and left thalamus (THdx, THsin) (Fig. 1). The placements of all ROIs were validated by an experienced neuroradiologist (IB).

### Statistical analysis

The statistical analysis was performed using Statistica 64 13.2 (Dell Inc., USA), except for the linear mixed-effects models for repeated measures, which were performed to investigate any difference between the right and left hemispheres where the Statistical Package for the Social Sciences (SPSS) was used. Descriptive statistics are expressed as the median and range. A Wilcoxon signed-rank test was used to evaluate motor and cognitive data before and after shunt surgery. The independent sample *t-*test was used for DTI parameters (FA and ADC) to investigate any difference between the iNPH and HI, and the dependent sample *t*-test was used for DTI parameters before and after surgery. Each pair of voxels was regarded as independent; therefore, no correction was made for multiple comparisons. The results were deemed to be significant at a value of p < 0.05 on Student’s t-test. Spearman’s correlation coefficient was used to estimate the relationship between motor function and DTI parameters.

## Results

### Clinical data

Thirteen iNPH patients underwent a shunt operation, and all of them showed significant improvement in their motor and balance function (w10mt, w10ms, TUG-t, TUG-s, Romberg) after surgery. However, their cognitive function, measured with the MMSE and TMT-A, was not improved significantly (Table 2). There was a significant difference between the patients and the HIs for w10mt, w10ms and Romberg but not for MMSE.

**Table 2:** Motor and cognitive results of iNPH patients (before and after shunt surgery) and HIs.

	w10mt (sec)	w10ms (steps)	TUG-t (sec)	TUG-s (steps)	MMSE	TMT-A (sec)	Romberg(sec)
iNPH pre-op median	16.1	26.4	20.7	28	27.7	64.3	24.5
3 months post-op median	11.4	19.9	15.3	20	27.9	62.8	46.9
Δ (pre-op vs. post-op)	4.7	6.5	5.4	8	0.2	1.5	22.4
p-value (pre-op vs. post-op)	**0.01**	**0.01**	**0.006**	**0.03**	0.57	0.79	**0.025**
HIs	7	10	N/A	N/A	29	N/A	60
p-value (pre-op vs. HIs)	0.05	0.008	N/A	N/A	0.08	N/A	0.02

pre-op: pre-operative patients; post-op: post-operative patients.

The motor and cognitive evaluation results are also displayed in Tables 1 and 2.

### Pre-operative DTI data vs. HIs DTI data

There was no significant difference between the right and left hemispheres. Therefore, we analysed both hemispheres together and compared the DTI data before the shunt operation with HIs’ DTI data. Two distinct patterns of FA were recognised. The first pattern was a higher FA in the patients than the HIs in the capsula interna, corpus callosum and thalamus. The other pattern was a lower FA in the frontal areas in the patients than in the HIs. However, more specifically, there was a significantly increased FA in the patients before the shunt operation in the splenium of the CC (p = 0.039) and the capsula interna (p = 0.01) and a significant decrease in the frontal white matter (p = 0.001). In the thalamus, there was a nearly significant (p = 0.07) increase compared to the HIs. For ADC, there was a significantly increased value in the frontal and lateral white matter (p = 0.001, p = 0.004) in the patients compared to the HIs. In the thalamus, there was an almost significant decrease in the patients compared to the HIs (p = 0.08) (Tables 3 and 4).

**Table 3:** FA values of pre- and post-operative patients and HIs, with statistical analysis.

ROIs for FA	CC genu	CC splenium	CI	CS	FWM	LWM	TH
Mean FA pre-op	0.65	0.77	0.64	0.4	0.29	0.355	0.36
Mean FA post-op	0.63	0.67	0.57	0.455	0.3	0.37	0.37
HIs	0.61	0.65	0.57	0.405	0.315	0.38	0.315
Difference post-op vs. pre-op	−0.02	−0.1	−0.07	0.055	0.01	0.015	0.01
Difference HIs vs. pre-op	−0.04	−0.12	−0.07	0.005	0.025	0.025	−0.045
Difference HIs vs. post-op	−0.04	−0.12	0	−0.05	0.015	−0.055	−0.055
p-value pre-op vs. post-op	0.74	**0.02**	**0.02**	0.4	0.3	0.61	0.44
p-value post-op vs. HIs	0.66	0.67	0.72	0.55	0.34	0.48	**0.02**
p-value pre-op vs. HIs	*0.08*	**0.039**	**0.01**	0.26	**0.02**	0.25	*0.07*

**Table 4:** ADC values of pre- and post-operative patients and HIs, with statistical analysis.

ROI for ADC	CC genu	CC splenium	CI	CS	FWM	LWM	TH
Mean ADC pre-op	991	893	711	757	910	859	776
Mean ADC post-op	944	960	730	771	868	862	796
Healthy individuals	1076	882	746	755	829	788	1008
Difference pre-op vs. post-op	47	−67	−19	−14	42	−3	−20
Difference HIs vs. pre-op	85	−11	35	−2	−81	−71	232
Difference HIs vs. post-op	132	−78	19	−16	−39	−74	212
p-value pre-op vs. post-op	0.38	0.09	0.11	0.34	***0.057***	0.92	0.22
p-value post-op vs. HIs	0.45	0.09	0.47	0.31	**0.04**	**0.03**	0.13
p-value pre-op vs. HIs	0.6	0.8	0.12	0.83	**0.001**	**0.047**	0.08

pre-op: pre-operative patients; post-op: post-operative patients.

### Post-operative DTI data vs. HIs’ DTI data

The differences between the patients and the HIs were generally small. The only significant difference was a post-operatively increased thalamic FA (p = 0.02) in the patients compared to the HIs. Regarding ADC, there was a significantly increased value in the frontal and lateral white matter in the patients post-operatively compared to the HIs (p = 0.04, p = 0.03) and a nearly significant p-value in the splenium of the CC (p = 0.09) (Tables 3 and 4).

### Pre-operative DTI data vs. post-operative DTI data

After surgery, there was a tendency towards normalisation of DTI parameters with a decrease in FA in the CC and capsula interna, whereas there was an increase in the frontal areas. Specifically, there was a significant FA decrease in the splenium of the CC (p = 0.02) and in the capsula interna (p = 0.02). Furthermore, we noticed a generally variable pattern for ADC but no significant changes. However, there was a trend towards a significant decrease in the frontal white matter (p = 0.057) (Tables 3 and 4).

### DTI data vs. clinical data

Spearman correlation coefficient statistics for all pre-operative DTI data showed a moderate to strong correlation, which was almost statistically significant, between ADC in lateral white matter and w10ms (r = 0.48, p = 0.09) and a strong significant correlation between ADC in the same area and w10mt (r = 0.68, p = 0.01). There was a significant strong and negative correlation between FA in the frontal white matter and w10ms (r = −0.7, p = 0.008) as well as for w10mt (r = −0.58, p = 0.04), a moderate but not significant correlation between FA in the thalamus and w10mt (r = 0.47, p = 0.1) and w10ms (r = 0.43, p = 0.14), and a moderate but not significant correlation between FA in the lateral white matter and w10mt (r = −0.46, p = 0.12) and w10ms (r = −0.27, p = 0.38) (Table 5).

**Table 5:** Correlation between gait results and DTI results in patients and HIs.

		ROI	w10mt (r)	p-value	w10ms (r)	p-value
PATIENTS	ADC	**CC genu**	0.18	0.55	0.14	0.65
		**CC splenium**	0.35	0.24	0.23	0.44
		**CI**	−0.35	0.24	−0.29	0.35
		**CS**	0.25	0.41	0.29	0.34
		**FWM**	0.13	0.66	0.07	0.81
		**LWM**	**0.68**	**0.01**	*0.48*	*0.09*
		**TH**	−0.21	0.49	−0.39	0.19
	**FA**	**CC genu**	−0.01	0.96	−0.07	0.83
		**CC splenium**	−0.32	0.29	−0.33	0.28
		**CI**	0.13	0.67	0.09	0.77
		**CS**	−0.37	0.21	−0.39	0.18
		**FWM**	**−0.58**	**0.04**	**−0.7**	**0.008**
		**LWM**	−0.46	0.12	−0.27	0.38
		**TH**	0.47	0.1	0.43	0.14
**HEALTHY GROUP**	**ADC**	**CC genu**	−0.3	0.44	0.05	0.89
		**CC splenium**	−0.33	0.39	0.02	0.97
		**CI**	−0.05	0.89	0.1	0.79
		**CS**	0.23	0.55	0.21	0.59
		**FWM**	0.14	0.72	*−0.6*	*0.08*
		**LWM**	−0.27	0.48	−0.6	0.56
		**TH**	0.5	0.17	−0.09	0.83
	**FA**	**CC genu**	−0.08	0.84	−0.19	0.63
		**CC splenium**	−0.1	0.8	−0.12	0.76
		**CI**	0.26	0.5	−0.45	0.23
		**CS**	−0.02	0.96	0.03	0.93
		**FWM**	**−0.67**	**0.04**	−0.66	0.055
		**LWM**	*−0.61*	*0.08*	−0.4	0.29
		**TH**	0.01	0.98	**−0.71**	**0.033**

Spearman correlation for HIs showed a strong significant correlation between FA (r = −0.67, p = 0.04) and w10ms in the frontal white matter and lateral frontal white matter (r = −0.61, p = 0.08) and a strong significant negative correlation between FA and w10mt in the thalamus area (r = −0.71, p = 0.03). For the ADC, there was a strong and almost significant negative correlation with w10mt in the frontal white matter (r = −0.6, p = 0.08) (Table 5).

Spearman correlations for changes in motor function before and after surgery versus changes in FA and ADC were also analysed, which revealed a strong and significant correlation between ΔFA and Δ10mt in the thalamus (r = 0.71, p = 0.007) and the frontal white matter area (r = −0.66, p = 0.01) and a strong significant correlation between ΔFA and Δ10ms in the thalamus (r = 0.58, p = 0.037) and the frontal white matter area (r = −0.68, p = 0.01). The other correlations were weak or absent. In the lateral frontal white matter area, there was an almost significant moderate correlation between ΔADC and Δ10ms (r = 0.51, p = 0.08) and a nearly significant moderate correlation between ΔADC and Δ10mt (r = 0.47, p = 0.1).

For the HIs, Spearman correlation showed a significant strong negative correlation between FA in the frontal white matter area and w10mt (r = −0.67, p = 0.04) and an almost significant negative correlation between FA in the same area and w10ms (r = −0.66, p = 0.055). The same pattern was found between FA in the lateral frontal white matter and w10mt (r−0.61, p = 0.08), although this difference was not statistically significant. There was a strong negative correlation between FA in the thalamus area and w10ms (r = −0.71, p = 0.033). A strong borderline significant negative correlation was found between ADC in the frontal white matter and w10ms (r = −0.6, p = 0.08).

## Discussion

Our study provides a clear indication of an ongoing pathological process in the periventricular white matter, especially in the thalamus and in the frontal white matter in iNPH patients, and shows that a shunt operation can partially reverse this process.

FA is a measure of microstructural integrity, and a low value is often seen in several neurological diseases and can be caused by axonal loss but may also be a result of stretch and oedema. A high FA may be caused by compression. ADC is a measure of the magnitude of diffusion of water molecules, and a high value may be indicative of oedema^17–20^.

Compared to the HIs, there were significantly higher FA values in the patients pre-operatively in the splenium of the CC and capsula interna and nearly significant FA values in the genu of the CC and thalamus. Furthermore, we noticed significantly lower FA in the frontal white matter area accompanied by significantly higher ADC (frontal and lateral frontal white matter area). In addition, we observed that the almost significantly higher FA in the thalamus area was accompanied by an almost significantly lower ADC in the thalamus area. A possible interpretation of these results is that in iNPH patients, there is a variable distributed compression of white matter in the different parts of the periventricular white matter depending partly on the proximity to the ventricular system and partly on the specific location. In the latter case, neurons may be more sensitive to pressure due to anatomical conditions.

Another possible interpretation would be that there is a more prominent oedema in frontal periventricular areas, but the results could also possibly be explained by axonal degeneration or a combination of these two states. Axonal degeneration could be caused by leucoaraiosis and microangiopathy since these also to some degree co-exist in these patients.

After shunt surgery, we found a trend towards the FA values of the HIs, i.e., normalisation in all areas but not in the thalamus. However, there were significantly lower FA values in the capsula interna and splenium of the CC but not in other investigated brain areas. For ADC, we could not find the same tendency except for a trend of a significant decrease in ADC in the frontal white matter area and an almost significant increase in the splenium of the CC. Hence, regarding FA in the capsula interna, both the results from the HIs vs. the patients and the decrease post-operatively support the notion that the capsula interna is more affected by compression without the requirement of excessive oedema. In the frontal white matter and lateral frontal white matter, the significantly decreased ADC in the HIs compared to the patients with a nearly significant decrease post-operatively could be interpreted as a diminished oedema.

The significant FA difference in the capsula interna and the CC splenium and the almost significant FA difference in the thalamus and the genu of the CC compared to HI are interesting findings but could be an epiphenomenon, and our results suggest more clearly FWM, the thalamus and their connections as key neuroanatomical areas where the pathogenesis takes place.

It is important to note that the patients all fulfilled the diagnostic criteria of probable iNPH, which makes this cohort a rather homogenous group in contrast to most previous DTI studies in iNPH^19,21^. All patients improved in their motor function after shunt surgery, whereas there was no evidence of cognitive improvement. However, one might argue that MMSE is a rather crude measure of cognition. This result adheres to the normal clinical situation of these patients, who tend to improve more in motor skills than in cognition^22^. Generally, the magnitude of improvement is very individual, and improvement is sometimes absent even if both clinical symptoms and radiological features suggest iNPH. A reasonable explanation would be that the impaired CSF circulation secondarily could lead to a degenerative process and that the neuroanatomical location is affected differently by pressure and distortion, giving a variable clinical response of a restored periventricular circulation.

Despite the fact that the HIs were slightly older than the patients, 75 vs. 77 years, we found significant results between iNPH patients and the HIs compared to several previous studies with younger HIs^19^.

The pre-operatively high FA results in CI seem to be an important finding that is in line with previous results from other research groups^19,23,24^. However, a problem is that patients with iNPH do not normally have any clinical signs of cortico-spinal involvement. The gait is more compatible with a higher level gait dysfunction in the frontal lobe^25^. Cognitive decline and urinary urgency/incontinence are also symptoms whose origin would be better explained by frontal lobe dysfunction, probably in the subcortical white matter. The thalamus and its wide connection to the frontal lobe is also a more plausible candidate for neuroanatomical correlate in the pathogenetic process^26–28^. These clinical considerations are supported by CBF studies showing a preponderant frontal lobe decreased blood flow^29,8,9^ but also a specific decreased blood flow in the thalamus,^30–32^. Support also comes from a previous magnetic resonance spectroscopy study from our group where we were able to demonstrate a significantly low level of N-acetyl aspartate in the thalamus^33^.

Regarding the patient group, we noticed a strong significant correlation between FA changes and gait results (time and steps) in the thalamus and a significant negative correlation between FA changes vs. gait results (time and steps) in the frontal white matter area. In the frontal lateral white matter, there was an almost significant correlation between the ADC change and gait results. For the thalamus, the greater the FA change, the better the gait was, and for the frontal white matter, the greater the FA changes and the lower the ADC changes, the worse the gait was. In our study, there was a significant gait improvement post-operatively, and we noticed increased FA and decreased ADC levels in frontal white matter, while both measures increased in the thalamus.

The correlation analysis of the HIs’ data showed a strong negative and significant association between FA and gait parameters (time and steps) in the frontal white matter area. Regarding ADC and gait results (steps), there was an almost statistically significant negative correlation in the frontal white matter area. This means that the higher the FA and ADC, the worse the gait. For the thalamus, we noticed a different pattern. There was a strong negative correlation between FA and gait (time) and a moderate, non-significant correlation between ADC and gait (time), meaning that the lower the FA and the higher ADC, the better the gait time was.

Correlating motor function with DTI data reveals interesting results that further support the hypothesis of thalamo-cortical (frontal) loop impairment. We noticed a rather strong correlation pre-operatively, r = −0.58 (p = 0.04) and r = −0.7 (p = 0.008), suggesting that the worse the motor function, the lower the FA in frontal white matter. Together with a significantly increased ADC in the frontal white matter and an almost significant change in ADC pre- vs. post-operatively (p = 0.057) would best be interpreted as a predominant oedema in the frontal white matter that subsides after surgery leading to a partial improvement because of co-existing irreversible axonal degeneration. In the capsula interna, the high FA decreased after shunt surgery, but there was no significant correlation in the FA change vs. the 10 m walk test. In the thalamus, the significantly higher FA in the patients vs. the His and a borderline significance for ADC (p = 0.08) are interesting findings supporting the view that the thalamus is an important site for the pathogenesis of iNPH. Correlation analysis in His showed the negative connection of FA with the gait steps in the thalamus r = −0.71 (p = 0.03) and in frontal areas r = 0.67 (p = 0.04) and the negative correlation between ADC and gait steps (but not for gait time) in frontal areas r = −0.6 (p = 0.08). The DTI vs. gait correlations among the patients and the His regarding the FA and ADC in frontal areas are similar, but the same is not true for the thalamus. This favours the interpretation that gait function is related to the subcortical frontal areas, but the discrepancy in regard to the thalamus might be due to insufficient power, as the p-values were 0.1 and 0.14 for the FA value.

Despite continuous research to understand the pathophysiological mechanism of ventriculomegaly and its influence on brain function, the aetiology of iNPH is still poorly understood. Vascular risk factors are overrepresented in patients with iNPH^34^. This is important to bear in mind since, white matter could be affected by microangiopathy. CBF studies have somewhat divergent results but indicate periventricular hypoperfusion mostly in the frontal lobe^8,31,35,36^. This is in line with the results of our DTI study, which raises the suspicion of both oedema and axonal degeneration in the periventricular area, presumably in the frontal area and in the thalamus. In studies focusing on neuropathological findings, oligodendrocyte loss, microglia activation and CC thinning in hydrocephalic rats have been described^37^. Other studies have referred to the existence of AD-related pathology in patients with iNPH^38–40^, and diminished clearance of toxic substances is yet another theory of its aetiology^41^.

According to previous DTI studies in iNPH, the majority of results have shown that FA was significantly lower in the area of the CC and significantly higher in the capsula interna than in the HIs. Our study shares these results, which strengthens its reliability. In the study by Keong and colleagues, there were significantly higher ADC values in hydrocephalic patients before shunt surgery compared to HIs but also a reduced FA in the capsula interna post-operatively, which is in line with our results^19^. In a study on AD and suspected NPH patients, DTI showed a significantly higher axial diffusivity in the capsula interna area^42^. Furthermore, in another DTI study, FA values were significantly lower in the iNPH group than in the HI group^24^. Abnormalities in the white matter have been reported with decreased FA in frontal, parietal, temporal, and supratentorial areas in the CC and capsula interna^11,23,24,42–53^.

### Limitations

First, the number of patients included in our study was small. Second, we performed this study with 1.5-tesla MR, whereas other studies used 3 T MR. Third, the ROIs were manually placed, which creates the disadvantage of subjectivity. Fourth, the ROI technique gives us the ability to approach the white matter area but may measure only a small area. This results in an average of the diffusion characteristics of a voxel and not of the entire multiple axonal tracts. This limitation is compensated by using 12 ROIs to identify white matter changes^54^. Finally, the existence of small artefacts from the shunt could possibly have influenced our results to some extent.

## Conclusions

We present significant and borderline-significant FA and ADC differences in the splenium, capsula interna, frontal subcortical areas and thalamus between HIs and iNPH patients, indicating a possible disease-related pathology. The changes between pre- and post-operative examinations suggest the reversibility of this pathology, resulting in improved motor function. The strong correlations between gait and DTI parameters of the thalamus and frontal white matter, both in the patients and in the HIs, as well as before and after shunt surgery, support the hypothesis of reversible thalamo-cortical circuit dysfunction in iNPH. In the future, larger DTI studies should focus on further evaluation of these findings in relation to pathophysiology and the utility of DTI in the diagnosis of iNPH.

## Supplementary information

Supplementary table 1.

Supplementary table 2.

Supplementary table 3.

Supplementary table 4.

Supplementary table 5.
