# Publisher Correction: Indication of Thalamo-Cortical Circuit Dysfunction in Idiopathic Normal Pressure Hydrocephalus: A Diffusion Tensor Imaging Study

**DOI:** 10.1038/s41598-020-69149-x

**Published:** 2020-07-16

**Authors:** Andreas Eleftheriou, Ida Blystad, Anders Tisell, Johan Gasslander, Fredrik Lundin

**Affiliations:** 1 Department of Neurology in Linköping, and Department of Biomedical and Clinical Sciences, Linköping University, Linköping, Sweden; 2 Department of Radiology in Linköping, and Department of Health, Medicine and Caring Sciences, Linköping University, Linköping, Sweden; 3 Department of Medical Radiation Physics, and Department of Health, Medicine and Caring Sciences, Linköping University, Linköping, Sweden; 4 Center for Medical Image Science and Visualisation (CMIV), Linköping University, Linköping, Sweden; 5 Department of Cardiology and Department of Health, Medicine and Caring Sciences, Linköping University, Norrkoping, Sweden

Correction to: *Scientific Reports* https://doi.org/10.1038/s41598-020-63238-7, published online 09 April 2020

The original version of this Article contained an error in the title of the paper, where the word “Diffusion” was missing. This has now been corrected in the PDF and HTML versions of the Article.

Additionally, as a result of production error, the tables in the main text were also included as supplementary files. The redundant supplementary files have now been removed from the Article.

