# Supplementary material for: Indication of Thalamo-Cortical Circuit Dysfunction in Idiopathic Normal Pressure Hydrocephalus: A Tensor Imaging Study
Source: Sci Rep. 2020 Apr 9;10:6148. doi: 10.1038/s41598-020-63238-7 (PMC7145806; doi:10.1038/s41598-020-63238-7)
Supplement: Supplementary file 1 — Supplementary table 1. [file 41598_2020_63238_MOESM1_ESM.docx]

| **DEMOGRAPHY AND COMORBIDITY** | | |
| --- | --- | --- |
| **CATEGORY** | **iNPH**  **(n=13)** | **HIs**  **(n= 9)** |
| ***Male/Female*** | 6/7 | 4/5 |
| ***Age, median (range), years*** | 75 (49-81) | 77 (70-90) |
| BMI (male/female) median | 27/28 | 23/21 |
| Diabetes Mellitus | 1 | 1 |
| Atrial Fibrillation | 1 | 1 |
| Hypertension | 6 | 1 |
| Claudicatio Intermittens | 1 | 0 |
| Stroke | 1 | 0 |
| Polyneuropathy | 2 | 0 |
| Heart disease | 4 | 0 |

Table 1: Clinical characteristics of the iNPH patients and HIs.

**Indication of Thalamo-Cortical Circuit Dysfunction in Idiopathic Normal Pressure Hydrocephalus:**

**A Diffusion Tensor Imaging Study**

**Andreas Eleftheriou^*a^, Ida Blystad^b^, Anders Tisell^c, d^, Johan Gasslander^e^, Fredrik Lundin^a^**

**^a^ Department of Neurology and Department of Clinical and Experimental Medicine, Linköping University, Linköping, Sweden**

**^b^ Department of Radiology, and Department of Medical and Health Sciences, Linköping University, Linköping, Sweden**

**^c^ Department of Radiation Physics, and Department of Medical and Health Sciences, Linköping University, Linköping, Sweden**

**^d^ Center for Medical Image Science and Visualisation (CMIV), Linköping University, Linköping, Sweden**

**^e^Department of Cardiology and Department of Health, Medicine and Caring Sciences, Linköping University, Norrkoping, Sweden**

**Andreas Eleftheriou (^*^corresponding author), M.D., Ph.D.c:** Department of Neurology, University Hospital, Linköping, Sweden , Garnisonsvägen 10, 58750, Linköping tel: +46733993945, fax: +46101032668 E-mail: 1) [andelef2002@yahoo.gr](mailto:andelef2002@yahoo.gr) and [Andreas.eleftheriou@regionostergotland.se](mailto:Andreas.eleftheriou@regionostergotland.se), ORCID:0000-0002-8535-1226
