# Supplementary material for: Indication of Thalamo-Cortical Circuit Dysfunction in Idiopathic Normal Pressure Hydrocephalus: A Tensor Imaging Study
Source: Sci Rep. 2020 Apr 9;10:6148. doi: 10.1038/s41598-020-63238-7 (PMC7145806; doi:10.1038/s41598-020-63238-7)
Supplement: Supplementary file 2 — Supplementary table 2. [file 41598_2020_63238_MOESM2_ESM.docx]

|  | **w10mt (sec)** | **w10ms (steps)** | | **TUG-t (sec)** | **TUG-s (steps)** | **MMSE** | | **TMT-A (sec)** | **Romberg**  **(sec)** |
| --- | --- | --- | --- | --- | --- | --- | --- | --- | --- |
| **iNPH-pre-op median** | 16.1 | 26.4 | | 20.7 | 28 | 27.7 | | 64.3 | 24.5 |
| **3 months post-op median** | 11.4 | 19.9 | | 15.3 | 20 | 27.9 | | 62.8 | 46.9 |
| **Δ-pre vs. post** | 4.7 | 6.5 | | 5.4 | 8 | 0.2 | | 1.5 | 22.4 |
| **p-value (pre-op vs. post-op)** | **0.01** | **0.01** | | **0.006** | **0.03** | 0.57 | | 0.79 | **0.025** |
| HIs | 7 | 10 | N/A | | N/A | 29 | N/A | | 60 |
| p-value (pre-op vs. HI) | 0.05 | 0.008 | N/A | | N/A | 0.08 | N/A | | 0.02 |

Table 2: Motor and cognitive results of iNPH patients (before and after shunt surgery) and HIs.

**Indication of Thalamo-Cortical Circuit Dysfunction in Idiopathic Normal Pressure Hydrocephalus:**

**A Diffusion Tensor Imaging Study**

**Andreas Eleftheriou^*a^, Ida Blystad^b^, Anders Tisell^c, d^, Johan Gasslander^e^, Fredrik Lundin^a^**

**^a^ Department of Neurology and Department of Clinical and Experimental Medicine, Linköping University, Linköping, Sweden**

**^b^ Department of Radiology, and Department of Medical and Health Sciences, Linköping University, Linköping, Sweden**

**^c^ Department of Radiation Physics, and Department of Medical and Health Sciences, Linköping University, Linköping, Sweden**

**^d^ Center for Medical Image Science and Visualisation (CMIV), Linköping University, Linköping, Sweden**

**^e^Department of Cardiology and Department of Health, Medicine and Caring Sciences, Linköping University, Norrkoping, Sweden**

**Andreas Eleftheriou (^*^corresponding author), M.D., Ph.D.c:** Department of Neurology, University Hospital, Linköping, Sweden , Garnisonsvägen 10, 58750, Linköping tel: +46733993945, fax: +46101032668 E-mail: 1) [andelef2002@yahoo.gr](mailto:andelef2002@yahoo.gr) and [Andreas.eleftheriou@regionostergotland.se](mailto:Andreas.eleftheriou@regionostergotland.se), ORCID:0000-0002-8535-1226
