# Supplementary material for: Indication of Thalamo-Cortical Circuit Dysfunction in Idiopathic Normal Pressure Hydrocephalus: A Tensor Imaging Study
Source: Sci Rep. 2020 Apr 9;10:6148. doi: 10.1038/s41598-020-63238-7 (PMC7145806; doi:10.1038/s41598-020-63238-7)
Supplement: Supplementary file 3 — Supplementary table 3. [file 41598_2020_63238_MOESM3_ESM.docx]

| **ROIs FA** | **CC genu** | **CC splenium** | **CI** | **CS** | **FWM** | **LWM** | **TH** |
| --- | --- | --- | --- | --- | --- | --- | --- |
| **Mean FA pre-op** | 0.65 | 0.77 | 0.64 | 0.4 | 0.29 | 0.355 | 0.36 |
| **Mean FA post-op** | 0.63 | 0.67 | 0.57 | 0.455 | 0.3 | 0.37 | 0.37 |
| **Healthy individuals** | 0.61 | 0.65 | 0.57 | 0.405 | 0.315 | 0.38 | 0.315 |
| **Difference post-op vs. pre-op** | -0.02 | -0.1 | -0.07 | 0.055 | 0.01 | 0.015 | 0.01 |
| **Difference HIs vs. pre-op** | -0.04 | -0.12 | -0.07 | 0.005 | 0.025 | 0.025 | -0.045 |
| **Difference His vs. post-op** | -0.04 | -0.12 | 0 | -0.05 | 0.015 | -0.055 | -0.055 |
| **p-value pre-op vs. post-op** | 0.74 | **0.02** | **0.02** | 0.4 | 0.3 | 0.61 | 0.44 |
| **p-value post-op vs. HIs** | 0.66 | 0.67 | 0.72 | 0.55 | 0.34 | 0.48 | **0.02** |
| **p-value pre-op vs. HIs** | *0.08* | **0.039** | **0.01** | 0.26 | **0.02** | 0.25 | *0.07* |

Table 3: Pre-, post-op and HIs FA values and statistical analysis.

**Indication of Thalamo-Cortical Circuit Dysfunction in Idiopathic Normal Pressure Hydrocephalus:**

**A Diffusion Tensor Imaging Study**

**Andreas Eleftheriou^*a^, Ida Blystad^b^, Anders Tisell^c, d^, Johan Gasslander^e^, Fredrik Lundin^a^**

**^a^ Department of Neurology and Department of Clinical and Experimental Medicine, Linköping University, Linköping, Sweden**

**^b^ Department of Radiology, and Department of Medical and Health Sciences, Linköping University, Linköping, Sweden**

**^c^ Department of Radiation Physics, and Department of Medical and Health Sciences, Linköping University, Linköping, Sweden**

**^d^ Center for Medical Image Science and Visualisation (CMIV), Linköping University, Linköping, Sweden**

**^e^Department of Cardiology and Department of Health, Medicine and Caring Sciences, Linköping University, Norrkoping, Sweden**

**Andreas Eleftheriou (^*^corresponding author), M.D., Ph.D.c:** Department of Neurology, University Hospital, Linköping, Sweden , Garnisonsvägen 10, 58750, Linköping tel: +46733993945, fax: +46101032668 E-mail: 1) [andelef2002@yahoo.gr](mailto:andelef2002@yahoo.gr) and [Andreas.eleftheriou@regionostergotland.se](mailto:Andreas.eleftheriou@regionostergotland.se), ORCID:0000-0002-8535-1226
