# Supplementary material for: Indication of Thalamo-Cortical Circuit Dysfunction in Idiopathic Normal Pressure Hydrocephalus: A Tensor Imaging Study
Source: Sci Rep. 2020 Apr 9;10:6148. doi: 10.1038/s41598-020-63238-7 (PMC7145806; doi:10.1038/s41598-020-63238-7)
Supplement: Supplementary file 4 — Supplementary table 4. [file 41598_2020_63238_MOESM4_ESM.docx]

| **ROIs ADC** | **CC genu** | **CC splenium** | **CI** | **CS** | **FWM** | **LWM** | **TH** |
| --- | --- | --- | --- | --- | --- | --- | --- |
| **Mean ADC pre-op** | 991 | 893 | 711 | 757 | 910 | 859 | 776 |
| **Mean ADC post-op** | 944 | 960 | 730 | 771 | 868 | 862 | 796 |
| **Healthy individuals** | 1076 | 882 | 746 | 755 | 829 | 788 | 1008 |
| **Difference pre-op vs. post-op** | 47 | -67 | -19 | -14 | 42 | -3 | -20 |
| **Difference HIs vs. pre-op** | 85 | -11 | 35 | -2 | -81 | -71 | 232 |
| **Difference His vs. post-op** | 132 | -78 | 19 | -16 | -39 | -74 | 212 |
| **p-value pre-op vs. post-op** | 0.38 | 0.09 | 0.11 | 0.34 | ***0.057*** | 0.92 | 0.22 |
| **p-value post-op vs. HIs** | 0.45 | 0.09 | 0.47 | 0.31 | **0.04** | **0.03** | 0.13 |
| **p-value pre-op vs. HIs** | 0.6 | 0.8 | 0.12 | 0.83 | **0.001** | **0.047** | 0.08 |

Table 4: Pre-, post-op and HIs ADC values and statistical analysis.

**Indication of Thalamo-Cortical Circuit Dysfunction in Idiopathic Normal Pressure Hydrocephalus:**

**A Diffusion Tensor Imaging Study**

**Andreas Eleftheriou^*a^, Ida Blystad^b^, Anders Tisell^c, d^, Johan Gasslander^e^, Fredrik Lundin^a^**

**^a^ Department of Neurology and Department of Clinical and Experimental Medicine, Linköping University, Linköping, Sweden**

**^b^ Department of Radiology, and Department of Medical and Health Sciences, Linköping University, Linköping, Sweden**

**^c^ Department of Radiation Physics, and Department of Medical and Health Sciences, Linköping University, Linköping, Sweden**

**^d^ Center for Medical Image Science and Visualisation (CMIV), Linköping University, Linköping, Sweden**

**^e^Department of Cardiology and Department of Health, Medicine and Caring Sciences, Linköping University, Norrkoping, Sweden**

**Andreas Eleftheriou (^*^corresponding author), M.D., Ph.D.c:** Department of Neurology, University Hospital, Linköping, Sweden , Garnisonsvägen 10, 58750, Linköping tel: +46733993945, fax: +46101032668 E-mail: 1) [andelef2002@yahoo.gr](mailto:andelef2002@yahoo.gr) and [Andreas.eleftheriou@regionostergotland.se](mailto:Andreas.eleftheriou@regionostergotland.se), ORCID:0000-0002-8535-1226
