# Supplementary material for: Indication of Thalamo-Cortical Circuit Dysfunction in Idiopathic Normal Pressure Hydrocephalus: A Tensor Imaging Study
Source: Sci Rep. 2020 Apr 9;10:6148. doi: 10.1038/s41598-020-63238-7 (PMC7145806; doi:10.1038/s41598-020-63238-7)
Supplement: Supplementary file 5 — Supplementary table 5. [file 41598_2020_63238_MOESM5_ESM.docx]

| **P**  **A**  **T**  **I**  **E**  **N**  **T**  **S** | **A**  **D**  **C** | **ROI** | **w10mt(r)** | **p-value** | **w10ms(r)** | **p-value** |
| --- | --- | --- | --- | --- | --- | --- |
|  |  | **CC genu** | 0.18 | 0.55 | 0.14 | 0.65 |
|  |  | **CC splenium** | 0.35 | 0.24 | 0.23 | 0.44 |
|  |  | **CI** | -0.35 | 0.24 | -0.29 | 0.35 |
|  |  | **CS** | 0.25 | 0.41 | 0.29 | 0.34 |
|  |  | **FWM** | 0.13 | 0.66 | 0.07 | 0.81 |
|  |  | **LWM** | **0.68** | **0.01** | *0.48* | *0.09* |
|  |  | **TH** | -0.21 | 0.49 | -0.39 | 0.19 |
|  | **F**  **A** | **CC genu** | -0.01 | 0.96 | -0.07 | 0.83 |
|  |  | **CC splenium** | -0.32 | 0.29 | -0.33 | 0.28 |
|  |  | **CI** | 0.13 | 0.67 | 0.09 | 0.77 |
|  |  | **CS** | -0.37 | 0.21 | -0.39 | 0.18 |
|  |  | **FWM** | **-0.58** | **0.04** | **-0.7** | **0.008** |
|  |  | **LWM** | -0.46 | 0.12 | -0.27 | 0.38 |
|  |  | **TH** | 0.47 | 0.1 | 0.43 | 0.14 |
| **H**  **E**  **A**  **L**  **T**  **H**  **Y**  **G**  **R**  **O**  **U**  **P** | **A**  **D**  **C** | **CC genu** | -0.3 | 0.44 | 0.05 | 0.89 |
|  |  | **CC splenium** | -0.33 | 0.39 | 0.02 | 0.97 |
|  |  | **CI** | -0.05 | 0.89 | 0.1 | 0.79 |
|  |  | **CS** | 0.23 | 0.55 | 0.21 | 0.59 |
|  |  | **FWM** | 0.14 | 0.72 | *-0.6* | *0.08* |
|  |  | **LWM** | -0.27 | 0.48 | -0.6 | 0.56 |
|  |  | **TH** | 0.5 | 0.17 | -0.09 | 0.83 |
|  | **F**  **A** | **CC genu** | -0.08 | 0.84 | -0.19 | 0.63 |
|  |  | **CC splenium** | -0.1 | 0.8 | -0.12 | 0.76 |
|  |  | **CI** | 0.26 | 0.5 | -0.45 | 0.23 |
|  |  | **CS** | -0.02 | 0.96 | 0.03 | 0.93 |
|  |  | **FWM** | **-0.67** | **0.04** | -0.66 | 0.055 |
|  |  | **LWM** | *-0.61* | *0.08* | -0.4 | 0.29 |
|  |  | **TH** | 0.01 | 0.98 | **-0.71** | **0.033** |

Table 5: Correlation between gait results and DTI results in patients and HIs.

**Indication of Thalamo-Cortical Circuit Dysfunction in Idiopathic Normal Pressure Hydrocephalus:**

**A Diffusion Tensor Imaging Study**

**Andreas Eleftheriou^*a^, Ida Blystad^b^, Anders Tisell^c, d^, Johan Gasslander^e^, Fredrik Lundin^a^**

**^a^ Department of Neurology and Department of Clinical and Experimental Medicine, Linköping University, Linköping, Sweden**

**^b^ Department of Radiology, and Department of Medical and Health Sciences, Linköping University, Linköping, Sweden**

**^c^ Department of Radiation Physics, and Department of Medical and Health Sciences, Linköping University, Linköping, Sweden**

**^d^ Center for Medical Image Science and Visualisation (CMIV), Linköping University, Linköping, Sweden**

**^e^Department of Cardiology and Department of Health, Medicine and Caring Sciences, Linköping University, Norrkoping, Sweden**

**Andreas Eleftheriou (^*^corresponding author), M.D., Ph.D.c:** Department of Neurology, University Hospital, Linköping, Sweden , Garnisonsvägen 10, 58750, Linköping tel: +46733993945, fax: +46101032668 E-mail: 1) [andelef2002@yahoo.gr](mailto:andelef2002@yahoo.gr) and [Andreas.eleftheriou@regionostergotland.se](mailto:Andreas.eleftheriou@regionostergotland.se), ORCID:0000-0002-8535-1226
